# Association between somatic *RET* mutations and clinical and genetic characteristics in patients with metastatic colorectal cancer

**DOI:** 10.1002/cam4.4400

**Published:** 2021-11-05

**Authors:** Yuan‐Zhong Yang, Wan‐Ming Hu, Liang‐Ping Xia, Wen‐Zhuo He

**Affiliations:** ^1^ Department of Pathology Sun Yat‐sen University Cancer Center State Key Laboratory of Oncology in South China Collaborative Innovation Center for Cancer Medicine Guangzhou Guangdong P. R. China; ^2^ VIP Region Sun Yat‐sen University Cancer Center State Key Laboratory of Oncology in South China Collaborative Innovation Center for Cancer Medicine Guangzhou Guangdong P. R. China

**Keywords:** colorectal cancer, mucinous histology, peritoneal metastasis, RET

## Abstract

**Background:**

Rearranged during transfection (*RET*) is a targetable oncogene. *RET* fusions have been reported in patients with metastatic colorectal cancer (mCRC). However, *RET* mutations in mCRC are less studied. Here, we aimed to characterize the clinical, pathological, and molecular landscape of *RET*‐mutated mCRC.

**Methods:**

Five hundred and eighty‐two patients were included in this study. Next‐generation sequencing was performed to detect *RET* mutations and calculate tumor mutation burden (TMB). We compared the clinical, pathological, and molecular characteristics of mCRC cases with tumors that harbored somatic *RET* mutations (*N* = 16, 2.7%) or had wild‐type *RET* (*N* = 566, 97.3%).

**Results:**

Males comprised the absolute majority of cases with *RET* mutations (15/16 [93.8%]) compared to their fraction among cases with wild‐type *RET* (339/566 [59.9%]). Furthermore, all patients with *RET* mutations were younger than 60 years (16/16 [100%]), whereas such patients were less predominant in the group with wild‐type *RET* (379/566 [67.0%]). Individuals with tumors positive for *RET* mutations more frequently exhibited mucinous histology (5/16 [31.2%] vs. 55/566 [9.7%]), exhibited a lower incidence of liver metastasis (4/16 [25.0%] vs. 335/566 [59.2%]), and higher incidence of peritoneal metastasis (9/16 [56.2%] vs.161/566 [28.4%]), expressed wild‐type *TP53* (8/16 [50.0%] vs.120/566 [21.2%]), and showed an increased frequency of MSI‐high (6/16 [37.5%] vs. 18/566 [3.2%]). In those with microsatellite‐stable mCRC, patients with *RET* mutations had a higher median TMB than patients with wild‐type *RET* (9.4 vs. 6.7 mutations/Mb, respectively, *p* = 0.001). The median progression‐free survival was similar in individuals with mutated and wild‐type *RET* on the oxaliplatin‐based regimen (7.1 vs. 8.7 months, *p* = 0.516).

**Conclusions:**

Our study suggests that cases with *RET* mutations represent a separate mCRC subtype. Further studies are needed to evaluate the efficacy of RET inhibitors in mCRC patients with *RET* mutations.

## Graphical abstract

RET mutations were enriched in male patients and those younger than 60 years.

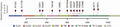

## 1 BACKGROUND

The *RET* (rearranged during transfection) gene encodes a receptor tyrosine kinase that plays a key role in the activation of MAPK, PI3K, JAK, and PKA/C pathways.^1^ RET signaling is essential for the normal development and function of kidney, nervous system, and hematopoiesis. Aberrant RET activation is involved in various types of tumorigenesis, including medullary thyroid cancer (MTC), non‐small cell lung cancer (NSCLC), and colorectal cancer (CRC).^2^, ^3^, ^4^ Thus, RET has emerged as a therapeutic target in patients with aberrant RET activation. Several multikinase inhibitors with RET inhibitory activity have been tested in clinic, although the efficacy is limited. Two selective RET inhibitors, selpercatinib (LOXO‐292) and pralsetinib (BLU‐667), have shown promising anticancer activities and are currently widely evaluated in clinical trials.^2^, ^5^, ^6^, ^7^, ^8^

There are mainly two mechanisms underlying *RET* oncogenic activation. The first is chromosomal rearrangement, which causes fusion of RET with a partner protein.^9^, ^10^ The second mechanism is somatic or germline gain‐of‐function mutation.^11^ The mutations could disrupt the intramolecular disulphide bonds and further transform the structure of extracellular domain. The mutant protein leads to the ligand‐independent dimerization, confers constitutive RET signal activation, and promotes cancer cell proliferation, migration, and growth. *RET* fusions were observed in a small fraction (<1%) of metastatic CRC (mCRC), where their presence was associated with old age, right‐sided tumor origin, wild‐type *RAS* and *BRAF*, microsatellite instability (MSI)‐high tumors, and poor prognosis.^12^ However, the prevalence as well as clinical, pathological, and molecular features of somatic *RET* mutations in patients with mCRC are largely unknown. Understanding the clinical impact of *RET* mutations can help oncologists to properly translate this information into clinical practice. Kato et al. examined a cohort of 300 patients with CRC and identified two individuals with *RET* mutations.^13^ Such rare occurrence of *RET* mutations precluded their further analysis. Hence, this study aimed to evaluate the frequency and phenotypic characteristics of mCRC with somatic *RET* mutation in a larger patient cohort.

## 2 METHODS

The following patients were selected: (1) those diagnosed with mCRC based on pathology samples at Sun Yat‐sen University Cancer between 1 January 2015 and 31 March 2020; (2) those who had an available record of the next‐generation sequencing result performed at our institute. Those who (1) accepted chemotherapy or radiotherapy before next‐generation sequencing analysis or (2) had no available follow‐up information were excluded. The study was performed in accordance with the Declaration of Helsinki and was approved by the Institutional Review Board of Sun Yat‐sen University Cancer (GZKJ2020‐02), and all patients provided informed consent. The detailed methods for next‐generation sequencing and tumor mutation burden (TMB) calculation are reported in a previously published paper^14^ and provided in the Supplementary Material.

We evaluated the association between the presence of somatic *RET* mutations and the following variables: age, gender, primary tumor location, differentiation, mucinous histology, T stage, N stage, time to metastasis, metastatic organs, *TP53* mutations, *APC* mutation, *RAS* mutations, *BRAF* mutations, *SMAD4* mutations, *PIK3CA* mutations, *ERBB2* amplifications, and MSI status. Patients’ characteristics were compared by using the Chi‐squared test. TMBs were compared by using the Kruskal–Wallis test. Kaplan–Meier survival analysis and log‐rank test were performed to detect differences in progression‐free survival (PFS). The statistical tests were two‐tailed and the differences were considered statistically significant when *p* < 0.05.

## 3 RESULTS

**FIGURE 1:**
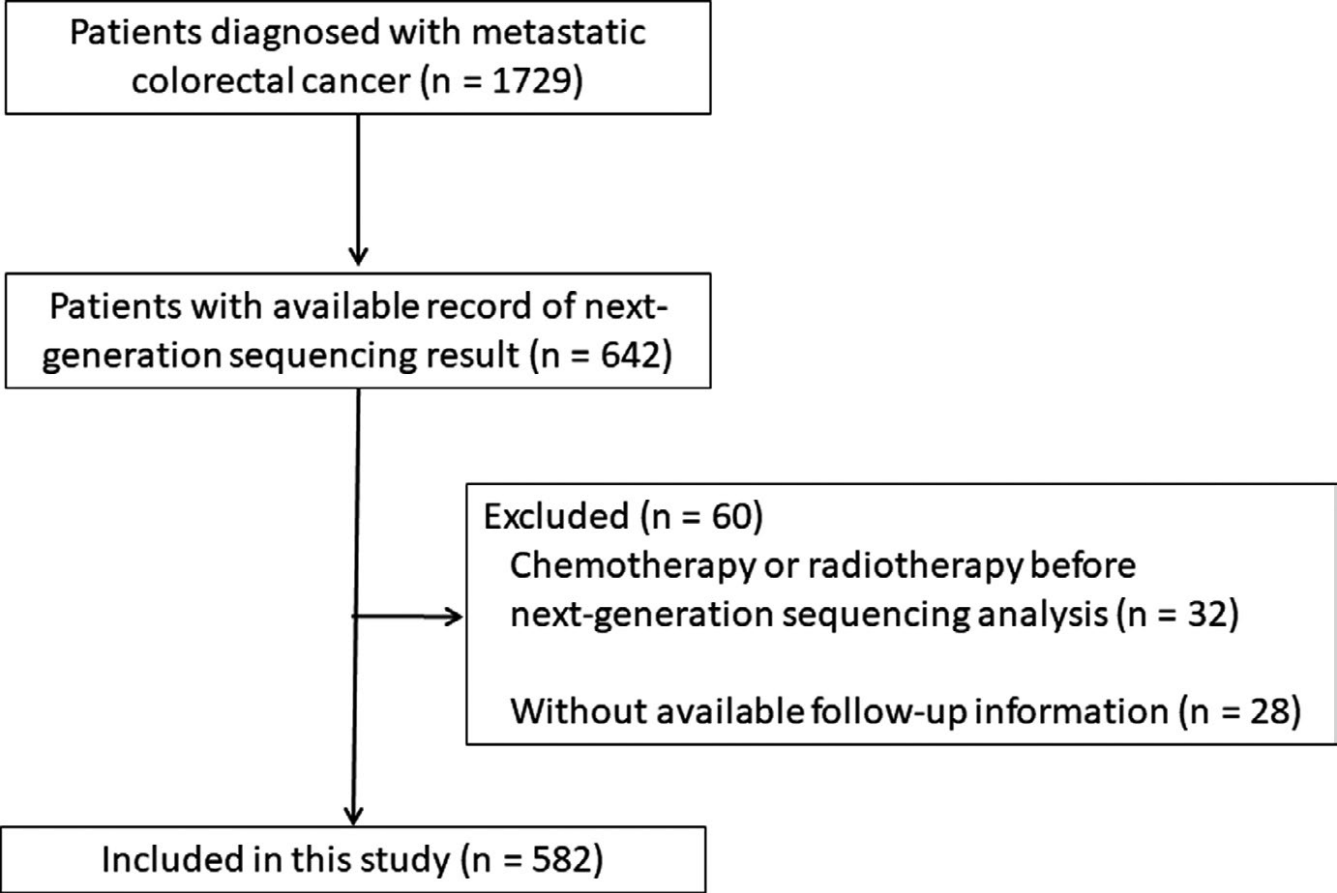
Flow chart describing patient selection for this study

**FIGURE 2:**
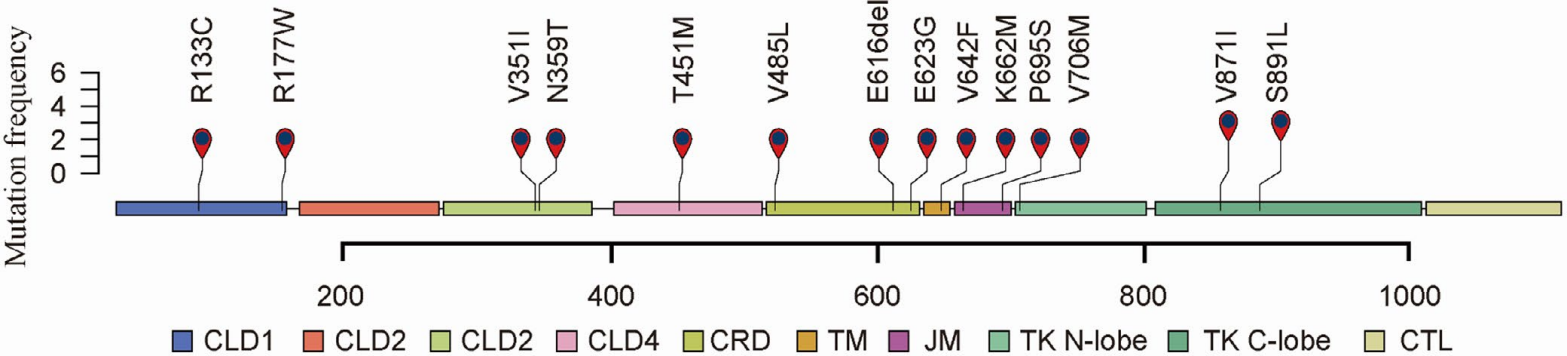
Frequent *RET* mutations detected in 582 patients with metastatic colorectal cancer

Five hundred and eighty‐two patients were included in this study (Figure 1). The patients’ median age was 54 (range, 17–88) years, and 354 patients (60.8%) were male. Somatic *RET* mutations were identified in 16 (2.7%) patients. The genomic alterations of *RET*‐mutated tumors are shown in Table 1 and Figure 2.

**TABLE 1:** Genomic alterations in *RET*‐mutated metastatic colorectal cancer

Patient ID	Genomic alterations
1	*RET*, EX3, R177W; *RAS* wild‐type; *BRAF* wild‐type; MSS
2	*RET*, EX3, R133C; *KRAS* A146T; *BRAF* wild‐type; MSS
3	*RET*, EX5, V351I; *RAS* wild‐type; *BRAF* wild‐type; MSI‐high
4	*RET*, EX6, N359T; *RAS* wild‐type; *BRAF* wild‐type; MSS
5	*RET*, EX7, T451M; *KRAS* G12D; *BRAF* wild‐type; MSS
6	*RET*, EX7, V485L; *NRAS* Q61L; *BRAF* wild‐type; MSI‐high
7	*RET*, EX10, E616del; *KRAS* G12D; *BRAF* wild‐type; MSI‐high
8	*RET*, EX10, E623G; *KRAS* G13D; *BRAF* wild‐type; MSS
9	*RET*, EX11, V642F; *RAS* wild‐type; *BRAF* wild‐type; MSS
10	*RET*, EX11, K662M; *RAS* wild‐type; *BRAF* wild‐type; MSS
11	*RET*, EX11, P695S; *KRAS* G13D; *BRAF* wild‐type; MSI‐high
12	*RET*, EX11, V706M; *RAS* wild‐type; *BRAF* wild‐type; MSS
13	*RET*, EX15, V871I; *RAS* wild‐type; *BRAF* wild‐type; MSI‐high
14	*RET*, EX15, V871I; *KRAS* G13D; *BRAF* wild‐type; MSI‐high
15	*RET*, EX15, S891L; *RAS* wild‐type; *BRAF* wild‐type; MSS
16	*RET*, EX15, S891L; *RAS* wild‐type; *BRAF* K601E; MSS

Abbreviations: MSI‐high: microsatellite instability‐high; MSS: microsatellite stable.

The presence of *RET* mutations was associated with male sex (15/16 [93.8%] patients with mutated *RET* vs. 339/566 [59.9%] patients with wild‐type *RET*), younger age (all 16 patients with mutated *RET* [100%] were ≤60 years old vs. 379 out of 566 [67.0%] patients with wild‐type *RET*), mucinous histology (5/16 [31.2%] patients with mutated *RET* vs. 55/566 [9.7%] patients with wild‐type *RET*), less liver metastasis (4/16 [25.0%] patients with mutated *RET* vs. 335/566 [59.2%] patients with wild‐type *RET*), and more peritoneal metastasis (9/16 [56.2%] patients with mutated *RET* vs. 161/566 [28.4%] patients with wild‐type *RET*, Table 2). Nine (12.3%) out of 73 male patients who were younger than 60 years of age and had peritoneal metastasis harbored *RET* mutations.

**TABLE 2:** Characteristics of patients with *RET*‐mutated metastatic colorectal cancer

Characteristics	*RET* ^mutation^, *N* (%)	*RET* ^wild‐type^, *N* (%)	*p*
Gender			
Male	15 (93.8)	339 (59.9)	**0.007**
Female	1 (6.2)	227 (40.1)	
Age			
>60	0 (0)	187 (33.0)	**0.002**
≤60	16 (100)	379 (67.0)	
Primary tumor location			
Right colon	6 (37.5)	162 (28.6)	0.603
Left colon	6 (37.5)	198 (35.0)	
Rectum	4 (25.0)	206 (36.4)	
Differentiation			
Well/moderate	11 (68.8)	416 (73.5)	0.784
Poor	5 (31.2)	150 (26.5)	
Mucinous histology			
Yes	5 (31.2)	55 (9.7)	**0.018**
No	11 (68.8)	511 (90.3)	
T stage			
1	0 (0)	2 (0.4)	0.415
2	0 (0)	21 (3.7)	
3	5 (31.2)	266 (47.0)	
4	7 (43.8)	161 (28.4)	
N stage			
0	6 (37.5)	123 (21.7)	**0.029**
1	0 (0)	164 (29.0)	
2	5 (31.2)	146 (25.8)	
Time to metastasis			
Synchronous	9 (56.2)	398 (70.3)	0.269
Metachronous	7 (43.8)	168 (29.7)	
Liver metastasis			
Yes	4 (25.0)	335 (59.2)	**0.009**
No	12 (75.0)	231 (40.8)	
Lung metastasis			
Yes	2 (12.5)	159 (28.1)	0.257
No	14 (87.5)	407 (71.9)	
Peritoneal metastasis			
Yes	9 (56.2)	161 (28.4)	**0.024**
No	7 (43.8)	405 (71.6)	
Distant nodes metastasis			
Yes	3 (18.8)	73 (12.9)	0.452
No	13 (81.2)	493 (87.1)	

Bold indicates statistical significance values *p* < 0.05.

As shown in Table 3, *RET* mutations were more frequent in patients with wild‐type *TP53* and MSI‐high tumors (6/16 [37.5%] patients with mutated *RET* vs. 18/566 [3.2%] patients with wild‐type *RET*). No Lynch syndrome was observed in patients with *RET* mutation. There were no significant associations between *RET* mutation presence and *APC*, *RAS*, *BRAF*, *SMAD4*, *PIK3CA*, or *ERBB2* status. In patients with microsatellite‐stable mCRC, those with *RET* mutations had a higher TMB than those with wild‐type *RET* (median TMB 9.4 and 6.7 Muts/Mb, respectively, *p* = 0.001). In patients with MSI‐high mCRC, those with *RET* mutations had a TMB similar to that of patients with wild‐type *RET* (median TMB 66.6 and 59.2 Muts/Mb, respectively, *p* = 0.923).

**TABLE 3:** Association between the presence of *RET* mutation and other molecular characteristics

Characteristics	*RET* ^mutation^, *N* (%)	*RET* ^wild‐type^, *N* (%)	*p*
*TP53*			
Wild‐type	8 (50.0)	120 (21.2)	**0.012**
Mutated	8 (50.0)	446 (78.8)	
*APC*			
Wild‐type	4 (25.0)	194 (34.3)	0.595
Mutated	12 (75.0)	372 (65.7)	
*RAS*			
Wild‐type	9 (56.2)	280 (49.5)	0.622
Mutated	7 (43.8)	286 (50.5)	
*BRAF*			
Wild‐type	15 (93.8)	503 (88.9)	0.319
V600E mutation	0 (0)	49 (8.7)	
No V600E mutation	1 (6.2)	14 (2.4)	
*SMAD4*			
Wild‐type	12 (75.0)	468 (82.7)	0.500
Mutated	4 (25.0)	98 (17.3)	
*PIK3CA*			
Wild‐type	13 (81.2)	481 (85.0)	0.721
Mutated	3 (18.8)	85 (15.0)	
*ERBB2*			
Amplification	16 (100)	548 (96.8)	1.000
No amplification	0 (0)	18 (3.2)	
MSI status			
MSS	10 (62.5)	548 (96.8)	**<0.001**
MSI‐high	6 (37.5)	18 (3.2)	

Bold indicates statistical significance values *p* < 0.05.

Abbreviations: MSI‐high, microsatellite instability‐high; MSS, microsatellite stable.

**FIGURE 3:**
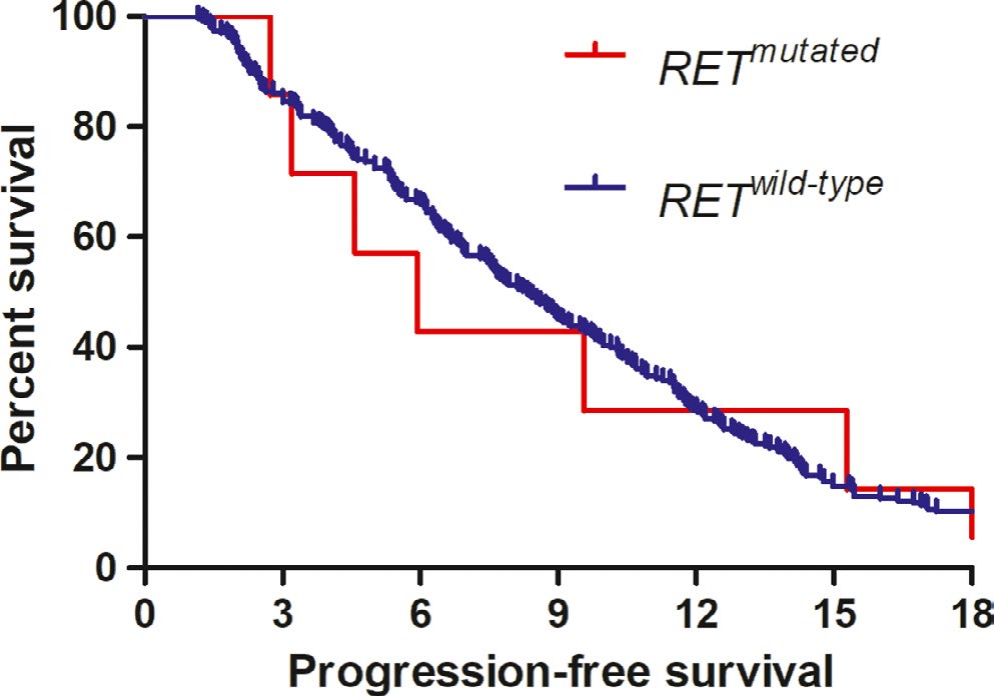
Comparison of progression‐free survival between patients with *RET* wild‐type and *RET*‐mutated tumors who were administered oxaliplatin‐based first‐line chemotherapy

The majority of the studied patients (413/582, 71.0%) were administered oxaliplatin‐based first‐line chemotherapy (FOLFOX or XELOX), whereas 63 (10.8%) received irinotecan‐based chemotherapy (FOLFIRI or XELIRI). Among the mCRC patients on the oxaliplatin‐based regimen, PFS was similar between patients with *RET* mutations and those with wild‐type *RET* (median PFS 7.1 vs. 8.7 months, respectively, *p* = 0.516, Figure 3).

## 4 DISCUSSION

In our analysis of 582 patients with mCRC, we detected *RET* mutations in 16 (2.7%) individuals. Furthermore, *RET* mutations were more often present in younger male patients and they were associated more strongly with mucinous histology, weaker liver metastasis, and stronger peritoneal metastasis. Given that the incidence of *RET* mutations in mCRC patients is low, our study provides valuable information to potentially help physicians identify the enriched subgroups in which *RET* mutations could be more frequent.

Kato et al. studied *RET* aberrations in 4,871 patients with diverse malignancies.^13^ Three hundred patients with CRC were included, and only two of them (0.7%) had *RET* mutations. The tumor stages were unknown, and this might explain the low mutation rate of *RET* in that study. Villava et al. analyzed 197 patients and identified four patients with colorectal cancer and *RET* mutation. Oliveira et al. also identified four different somatic *RET* mutations in patients with colon cancer. However, the rare occurrence of *RET* mutations precluded further analysis in those studies. Recently, Zhao et al. retrospectively reviewed 3,272 patients with CRC and documented that the *RET* mutation rate was 3.39%,^11^ which was similar to the rate observed in the present study. *RET* fusions have been observed in a small subgroup of patients with mCRC. *RET* fusions were associated with older age, origin on the right side, wild‐type *RAS* and *BRAF*, MSI‐high tumors, as well as shorter survival.^12^ Our study makes several contributions to the current literature. First, we found the association between *RET* mutations and patients’ characteristics (sex, age, histology, and metastasis sites). These additional information revealed in our study suggests a potential enrichment strategy for further trials with targeted agents focused on the *RET* mutation. Second, we found *RET* mutations were enriched in younger and male patients. We did not observe significant correlations between the presence of *RET* mutations and primary tumor location and *RAS* or *BRAF* status, on the other hand. These results suggest that factors affecting the incidence of *RET* mutations are different from those associated with *RET* rearrangements, and *RET*‐mutated tumors did not share with *RET* fusions the same clinical and pathological characteristics. In our study, MSI‐high was associated with *RET* mutation. This may be caused by genome instability in MSI‐high tumors. Further studies are needed to evaluate the impact of *RET* mutation on response to immunotherapy in MSI‐high tumors.

To date, there are several commercially available inhibitors demonstrating activity against RET. Most of them are nonspecific inhibitors, such as cabozantinib, vandetanib, lenvatinib, sorafenib, sunitinib, and alectinib.^15^ Recently, two selective RET inhibitors have been introduced to the clinic. Pralsetinib has been demonstrated to inhibit the growth of tumors driven by various *RET* mutations and fusions in vivo, and it showed durable clinical responses in patients with *RET*‐altered NSCLC.^16^, ^17^ Selpercatinib also showed promising activity in patients with *RET*‐fused NSCLC and RET‐fused or mutated MTC.^7^, ^8^, ^15^, ^18^ Two patients with *RET*‐mutated mCRC were included in a clinical trial (NCT03037385), and the efficacy of pralsetinib is still under evaluation in these two patients.

Our study had few limitations, such as a limited sample size and retrospective bias. The identified *RET* mutations in this study could be driver aberrations or only neutral passengers. We could not distinguish between them owing to limited information regarding *RET* mutations in mCRC. Furthermore, because the follow‐up time was limited, overall survival could not be determined.

## 5 CONCLUSIONS

Despite these limitations, our study noted *RET* mutation in 2.7% patients with mCRC, and showed males younger than 60 years of age comprised the absolute majority of patients with *RET* mutations. These observations suggest that tumors with *RET* mutation represent a novel subtype of mCRC, and are different from tumors with *RET* fusion. Further studies are needed to evaluate the efficacy of RET inhibitors in patients with *RET*‐mutated mCRC.

## ETHICS STATEMENT

All patients signed informed consent. The study was performed in accordance with the Declaration of Helsinki and was approved by the Institutional Review Board of Sun Yat‐sen University Cancer Center (GZKJ2020‐02).

## CONFLICT OF INTEREST

The authors declare that they have no competing interest.

## AUTHOR CONTRIBUTION

Concept and design: All authors. Acquisition, analysis, or interpretation of data: YZ Yang and WM Hu. Drafting of the manuscript: WZ He and LP Xia. Critical revision of the manuscript for important intellectual content: All authors. Statistical analysis: WZ He, WM Hu, and LP Xia. Obtained funding: WZ He. Administrative, technical, or material support: YZ Yang and WM Hu. Supervision: LP Xia.

## Supporting information

Supplementary Material

## Data Availability

The sequence data generated and/or analyzed during the current study are not publicly available [Data are stored in a closed system in the hospital] but are available from the corresponding author upon reasonable request. The clinical data analyzed during the current study are available in the Research Data Deposit system of Sun Yat‐sen University Cancer [RDDA2021966801, https://www.researchdata.org.cn/default.aspx].
