# Supplementary material for: Association between somatic RET mutations and clinical and genetic characteristics in patients with metastatic colorectal cancer
Source: Cancer Med. 2021 Nov 5;10(24):8876–82. doi: 10.1002/cam4.4400 (PMC8683541; doi:10.1002/cam4.4400)
Supplement: Supplementary file 1 — Supplementary Material [file CAM4-10-8876-s001.docx]

**Sample processing and DNA extraction**

Tumor tissue were sampled via needle aspiration. Formalin fixation and paraffin embedding were then performed, followed by histologic section preparation. All PB samples were collected in cell-free DNA blood collection tubes (Streck, Omaha, NE, USA) at room temperature, and would be processed within 72 h of collection. Plasma was separated by centrifugation at 2,500 × g for 10 min, transferred to microcentrifuge tubes, and centrifuged at 16,000 × g for 10 min to remove cell debris. Peripheral blood lymphocytes (PBL) were isolated from cell pellets following the initial spin. Genomic and germline DNA was extracted from tumor tissue and PBLs using the DNeasy Blood & Tissue Kit (Qiagen, Hilden, Germany). Circulating cell-free DNA (cfDNA) was extracted from 0.6-1.8 ml plasma using the QIAamp Circulating Nucleic Acid Kit (Qiagen). All DNA was quantified using a Qubit fluorometer (Invitrogen, Carlsbad, CA, USA), and cfDNA fragment length were assessed using the Agilent 2100 BioAnalyzer (Agilent Technologies, Santa Clara, CA, USA).

**Library construction and hybridization capture-based sequencing**

Library construction and hybridization capture-based sequencing were performed as previously reported[1]. Tissue and PBL DNA was sheared to 200-250 bp fragments with a Covaris S2 instrument (Woburn, MA, USA) before library construction. After end-repair and polyA tailing, adapters with unique identifiers were ligated to both ends of double-stranded cfDNA fragments. Indexed Illumina NGS libraries were prepared from tissue, PBL germline and cfDNA using the KAPA Library Preparation Kit (Kapa Biosystems, Boston, MA, USA). The SeqCap EZ Library system (Roche NimbleGen, Madison, WI, USA) was used for target enrichment. All libraries were hybridized to custom-designed biotinylated oligonucleotide probes (IDT, Coralville, IA, USA) covering 1.09 Mbp of the genome. The captured genomic regions included the most common driver genes of solid tumors, including CRC. We chose their entire exome regions to construct the basic panel. Next, genomic regions relevant to the effects of chemotherapy, targeted drugs, and immunotherapy per available clinical and pre-clinical research were added to the panel. Finally, high-frequently mutant regions recorded in the Catalogue of Somatic Mutations in Cancer (COSMIC, http://cancer.sanger.ac.uk/cosmic) and The Cancer Genome Atlas (TCGA, https://cancergenome.nih.gov/) were involved. All included 1017 genes are shown in Table S1. Capture hybridization was performed per the manufacturer’s protocol (Illumina, San Diego, CA, USA). Captured DNA fragments were amplified after hybrid selection and then pooled into several multiplexed libraries. Sequencing was performed using the Illumina HiSeq 3000 or NextSeq CN500 instruments with 75 × 75 paired-end reads per the manufacturer’s recommendations using Illumina’s TruSeq PE Cluster Generation Kit v3 and the TruSeq SBS Kit v3.

**Raw data processing**

After removal of terminal adaptor sequences and low-quality reads (>50% N rate, >50% bases with Q<5), remaining reads were mapped to the reference human genome (hg19) and aligned using Burrows-Wheel Aligner (http://bio-bwa.sourceforge.net/) with default parameters, followed by duplicate reads identification using Picard’s Mark Duplicates tool (https://software.broadinstitute.org/gatk/documentation/tooldocs/4.0.3.0/picard_sam_markduplicates_MarkDuplicates.php).

Base quality recalibration and local realignment were conducted by the Gene Analysis Toolkit (GATK, https://www.broadinstitute.org/gatk/). The median depth for 152 blood samples were 3576×, and the range was from 883 to 8785×. As for 12 tissue samples, the median depth was 1554.5× (range from 883 to 2298×)[1].

**Mutation identification**

Somatic insertions/deletions (indels) and single nucleotide variants (SNVs) were identified with the GATK and MuTect2 software. After mutation calling, several filter procedures would be executed: (1) Germline mutations were not analyzed in this study, and were filtered by matched PBL data. (2) Synonymous variants were filtered. (3) Variants with less than 5 high-quality sequencing reads (mapqthres>30, baseqthres>30) were removed. (4) We had built an inhouse database involving blood sequencing results from about 1000 healthy subjects. Variants were filtered as cross-contamination if present in >1% samples in custom single nucleotide polymorphism (SNP) databases and self-built SNP database. These filtered variants were identified as high-confidence somatic mutations[1].

**Reference for supplementary method**

1. Cao W, Xu Y, Chang L, Gong Y, Li L, Mo X, Zhang X, Lin G, Zhou J, Liu D *et al*: **Genotyping of Circulating Tumor DNA Reveals the Clinically Actionable Mutation Landscape of Advanced Colorectal Cancer**. *Mol Cancer Ther* 2019, **18**(6):1158-1167.
